# The Changing Landscape of Advanced Drug Delivery: From Passive Carriers to Intelligent Therapeutic Platforms

**DOI:** 10.3390/pharmaceutics18070835

**Published:** 2026-07-08

**Authors:** Milena A. Vega, Carlos Alonso

**Affiliations:** 1 Departamento de Ingeniería Química y Textil, Facultad de Ciencias Químicas, Universidad de Salamanca, 37008 Salamanca, Spain; c.alonso@usal.es; 2 Instituto de Investigación Biomédica de Salamanca (IBSAL), Complejo Asistencial de Salamanca, Paseo de San Vicente, 58, 37008 Salamanca, Spain

## 1. The Evolution of Drug Delivery Systems

The development of drug delivery systems (DDSs) has traditionally been driven by the need to overcome well-recognized pharmacokinetic limitations, including poor aqueous solubility, rapid degradation of therapeutic agents, low bioavailability, and short circulation times [1,2]. The emergence of liposomes, polymeric nanoparticles (NPs), nanoemulsions, and other nanostructured carriers represented an important advance, allowing numerous compounds to be delivered more efficiently while improving their stability and therapeutic performance [3,4]. As the field has progressed, the objectives of DDSs have also expanded. Current research focuses on the development of platforms capable of interacting with biological environments, responding to local physiological or pathological conditions, and regulating therapeutic activity according to the characteristics of the target tissue [5,6].

This transformation has been driven by the convergence of materials science, pharmaceutical technology, chemistry, molecular biology, engineering, and medicine. Progress is therefore no longer determined exclusively by the discovery of new nanomaterials but by the ability to integrate complementary biological functions into systems that remain reproducible, scalable, and suitable for clinical translation. In this context, the distinction between a material and a therapeutic platform is becoming increasingly blurred, as nanocarriers are designed not only to transport drugs but also to participate actively in the therapeutic process [7].

The studies collected in this Special Issue reflect the present direction of the field. Although they encompass different classes of materials and address distinct biomedical challenges, they converge on a common objective: developing DDSs capable of interacting more effectively with biological systems while overcoming limitations that remain difficult to address using conventional formulations. Collectively, the studies offer a representative overview of several recent research directions that continue to shape the evolution of pharmaceutical nanotechnology. These emerging trends are discussed in the following sections.

## 2. From Passive Carriers to Multifunctional Therapeutic Platforms

One of the most noticeable developments in advanced drug delivery (ADD) research is the progressive transition from systems designed to perform a single function toward platforms capable of combining multiple complementary properties. While improving drug stability or prolonging circulation time remains important, these objectives are increasingly combined with active targeting, controlled drug release, responsiveness to biological stimuli, enhanced cellular uptake, and improved biocompatibility. As a result, the performance of a DDS is no longer determined solely by its ability to transport a therapeutic agent but by how effectively it interacts with the biological environment throughout the therapeutic process [1,8].

This tendency is well represented by Contribution 1, where the development of stimuli-responsive cationic lyotropic liquid crystalline nanoparticles (NPs) demonstrates how the rational design of nanostructure architecture can influence several properties simultaneously. The combination of a highly organized internal structure, pH responsiveness, strong positive surface charge, excellent colloidal stability, and high drug-loading capacity illustrates how a single formulation can integrate characteristics that collectively improve its therapeutic potential rather than optimizing only one individual parameter. Such systems exemplify the increasing importance of designing nanocarriers whose physicochemical properties remain closely linked to their biological performance.

A different example of multifunctionality is provided by Contribution 2, where natural biopolymers are combined to improve the therapeutic performance of quercetin against multidrug-resistant Klebsiella pneumoniae. In this case, nanoencapsulation not only protects the active compound and enables sustained drug release but also enhances antibacterial activity, improves biofilm inhibition and eradication, and maintains excellent biocompatibility. Rather than acting exclusively as a delivery vehicle, the nanosystem contributes directly to the therapeutic outcome by combining several complementary functions within the same formulation. This strategy illustrates how multifunctionality has become equally relevant beyond oncology, particularly in the growing effort to combat antimicrobial resistance.

In addition to combining multiple physicochemical properties, modern DDSs now incorporate molecular recognition mechanisms capable of directing therapeutic agents toward specific cell populations. This concept is illustrated by Contribution 3, where magnetic NPs functionalized with DNA aptamers combine active targeting with pH-responsive drug release for hepatocellular carcinoma treatment. The incorporation of aptamers enhances nanoparticle (NP) internalization by tumor cells while maintaining low interaction with healthy cells, demonstrating how surface functionalization can substantially improve treatment selectivity. Together with the preferential release of sorafenib under acidic conditions, this platform reflects the growing interest in exploiting the biological characteristics of diseased tissues to improve therapeutic precision while minimizing off-target effects.

A complementary perspective emerges from biological systems themselves. As materials science continues to expand the complexity of DDSs, greater attention is also being paid to biological mechanisms capable of inspiring the design of new therapeutic platforms.

Contribution 4 explores this concept by examining the role of sialic acid in neurodegenerative and psychiatric disorders. Beyond its structural role in glycoconjugates, sialic acid participates in neuronal communication, immune regulation, and blood–brain barrier function. These biological properties are now being explored to develop nanocarriers capable of exploiting endogenous recognition mechanisms to improve brain-targeted drug delivery. Rather than relying exclusively on synthetic modifications to overcome biological barriers, this strategy seeks to harness naturally occurring physiological processes to facilitate selective drug transport and tissue recognition.

Although these contributions differ considerably in composition, therapeutic application, and disease model, they collectively illustrate a common direction in pharmaceutical nanotechnology. DDSs are redefining themselves from passive carriers toward adaptive therapeutic platforms capable of combining structural design, biological responsiveness, and molecular recognition within a single formulation. This convergence of material engineering and biological understanding is gradually redefining how nanocarriers are conceived and will likely continue shaping the next generation of advanced therapeutic systems.

## 3. From Disease-Specific Formulations to Adaptable Therapeutic Platforms

An important consequence of this transformation is the versatility that modern DDSs have acquired. Although pharmaceutical nanotechnology has traditionally been associated with cancer therapy, many of the engineering principles that initially emerged within oncology are now being successfully translated to fields as diverse as infectious diseases, regenerative medicine, cardiovascular medicine, and disorders of the central nervous system [9,10]. 

Their broad applicability suggests a future in which versatile platforms can be adapted to different biological contexts without the need to develop entirely new systems for every therapeutic indication [11].

Despite the diversity of existing clinical applications being explored, several common objectives can be identified. Improving treatment selectivity, preserving drug stability, controlling drug release, enhancing interactions with target tissues, and reducing systemic toxicity have become shared design principles regardless of the therapeutic indication. In many cases, differences among biomedical applications arise from the biological characteristics of each disease rather than from the engineering concepts used to design the delivery system itself [1,12].

This growing versatility also reflects a gradual convergence between disciplines that until recently evolved largely independently. Advances in oncology are now influencing the development of antimicrobial therapies, while knowledge generated in neuroscience contributes to improving targeting strategies for other pathological conditions. At the same time, progress in biomaterials, polymer chemistry, and molecular biology continues to provide new opportunities for adapting existing technologies and an expanding range of therapeutic challenges [1,2].

Today’s efforts focus on modular systems whose composition, surface chemistry, or targeting ligands can be adapted according to specific therapeutic requirements. Such flexibility not only broadens the potential applications of existing technologies but also facilitates the transfer of knowledge between different biomedical disciplines, accelerating the development of new therapeutic solutions [1,13].

This ability to transfer design principles across different therapeutic areas may become one of the defining characteristics of the next generation of DDSs. Instead of producing isolated technological solutions, future research will increasingly focus on adaptable therapeutic platforms whose biological performance can be tailored through relatively simple modifications in composition, surface functionalization, or molecular targeting. As these technologies continue to evolve, the boundaries separating different biomedical fields will likely become less pronounced, allowing innovations developed for one application to accelerate progress across many others [1,14].

## 4. From Experimental Platforms to Clinical Practice

Pharmaceutical nanotechnology has advanced rapidly during the last two decades, expanding the possibilities of drug delivery far beyond what was considered feasible only a few years ago. New materials, improved manufacturing strategies, and a deeper understanding of biological systems have broadened the range of therapeutic platforms available for development. Nevertheless, relatively few nanomedicines have successfully reached routine clinical practice. This difference between experimental progress and clinical implementation continues to shape the present landscape of the field [15].

Excellent experimental performance alone is rarely sufficient for successful clinical translation. A formulation that performs well under controlled laboratory conditions must also demonstrate reproducible manufacturing, long-term physicochemical stability, acceptable production costs, and compatibility with regulatory requirements before it can become a therapeutic product. As delivery systems incorporate additional functionalities, these requirements become progressively more demanding. Targeting ligands, stimulus-responsive components, imaging capabilities, and combination therapies offer clear therapeutic advantages; however, they also increase the complexity of manufacturing, quality control, and large-scale production [1,2].

Biological systems introduce another level of variability that remains difficult to reproduce experimentally. Immediately after administration, NPs interact with proteins, immune cells, extracellular matrices, and numerous biomolecules that influence their stability, biodistribution, cellular uptake, and therapeutic activity. These interactions frequently modify NP behavior in ways that cannot be fully anticipated using conventional in vitro models. Consequently, positive experimental outcomes do not always translate into comparable clinical performance [16].

This situation has encouraged the development of experimental models that better reproduce the complexity of human physiology. Three-dimensional cell cultures, organ-on-chip technologies, patient-derived models, computational simulations, and artificial intelligence are providing new opportunities to evaluate DDSs under conditions that more closely resemble the biological environment encountered in patients. Their incorporation into preclinical research should improve the selection of formulations with a higher probability of successful clinical evaluation while reducing the gap between laboratory observations and clinical outcomes [17,18].

Progress will also depend on closer collaboration between disciplines that have traditionally evolved along separate paths. Materials scientists, pharmaceutical researchers, chemists, engineers, biologists, clinicians, and regulatory specialists all contribute different perspectives to the development of drug delivery technologies. Integrating this expertise from the earliest stages of research may help identify limitations that could otherwise compromise later phases of development, facilitating a more efficient transition from experimental design to clinical application [1,2].

The long-term impact of pharmaceutical nanotechnology will be determined as much by successful clinical translation as by scientific innovation. Expanding the functional capabilities of DDSs remains an important objective, yet equal attention must be given to reproducibility, scalability, manufacturing, regulatory requirements, and long-term safety. Bringing these elements together will determine which of the therapeutic platforms currently under development successfully move from experimental concepts to technologies capable of improving patient care [5,6].

## 5. Continuing the Scientific Dialog

The studies brought together in this Special Issue reflect the current state, which is still evolving thanks to the contributions of researchers with diverse scientific backgrounds. Although each contribution addresses a specific question, together they illustrate the breadth of current research and the opportunities created by the growing combination of material design and biological understanding. Continued collaboration across these fields will remain essential for transforming scientific advances into future therapeutic solutions.

The ideas presented throughout this Special Issue also provide a starting point for future research. To continue this scientific dialog, a second edition of the Special Issue, Advanced Nanomaterials for Drug Delivery Systems and Pharmaceutical Applications, has recently been launched. Building on the work presented in the current volume, the new edition will welcome contributions addressing intelligent biomaterials, targeted drug delivery, stimulus-responsive systems, regenerative medicine, nanomedicine, and other emerging topics that continue to shape pharmaceutical nanotechnology. We hope it will provide a forum for exchanging ideas, strengthening collaborations, and promoting further advances in drug delivery research.

## 6. Conclusions

Drug delivery research has expanded far beyond its original objective of improving drug transport. Today, advances in material design, biology, and pharmaceutical technology are converging to create therapeutic platforms capable of interacting with complex biological systems in more selective ways. This transition is opening new possibilities across a wide range of biomedical applications thereby redefining the role of nanotechnology in modern therapeutics.

The studies presented in this Special Issue capture one stage in this continuing development. Together, they reflect the diversity of ideas currently shaping the field and illustrate how scientific progress continues to emerge from the close interaction between disciplines that were once considered largely independent.
